# Geopolitical risks and the spillover of food market risks: case analysis of the Chinese grain market

**DOI:** 10.3389/fnut.2026.1924317

**Published:** 2026-08-31

**Authors:** Jing Li, Yue Yang

**Affiliations:** 1 School of Politics and International Relations, Lanzhou University, Lanzhou, China; 2 School of Economics and Management, China University of Geosciences (Wuhan), Wuhan, China

**Keywords:** food safety, geopolitical risk, grain price shock, main grain crop, market risk

## Abstract

Maintaining the stability of food prices is crucial for achieving sustainable development in grain markets, particularly in the context of evolving international conditions and geopolitical risks. This study employs the DCC-GARCH model to examine the dynamic relationship between international and domestic grain prices, focusing on the price stability of major staple crops: soybeans, wheat, and corn. By applying generalized forecast error variance decomposition, we quantify the risk spillover effects within grain markets and clarify the role of geopolitical uncertainties in shaping such spillovers. The results reveal a pronounced time-varying pattern in the transmission of international grain prices to domestic prices, with discernible differences across crop types. Within-market risk spillovers significantly outweigh cross-market spillovers, identifying the international grain market as the primary conduit for risk transmission. Geopolitical uncertainty exerts short-term negative shocks on grain market risk spillovers and on the net spillovers of both international and domestic soybeans. Simultaneously, an increase is observed in the net spillover levels of both domestic and international wheat markets. The magnitude of geopolitical risk impacts on grain markets varies across distinct periods, with the Russia-Ukraine conflict exerting a temporary negative influence. Taking the 2022 Russia-Ukraine conflict and the Sino-US trade friction as typical cases, it further verified that geopolitical risks pose a substantive threat to China’s food import security through two channels: directly impacting import sources and indirectly increasing production costs. Amid international trade uncertainties and geopolitical pressures, adopting innovative measures to bolster food security and improve nutrition is essential for advancing Sustainable Development Goal 2 (Zero Hunger). These findings offer significant implications for enhancing the stability of supply chains and market prices for key grains, particularly for agricultural importing countries.

## 1 Introduction

Food serves as an essential foundation for human survival and societal development. Good agricultural output is of great significance for maintaining the sustainable development of the economy (1, 2). Food security, as commonly defined, extends beyond national self-sufficiency to encompass all citizens’ physical and economic access to sufficient, safe, and nutritious food that meets their dietary needs and food preferences for an active and healthy life (3, 4). Promoting the sustainable development of grain markets is a vital component of achieving Sustainable Development Goal 2 (Zero Hunger). Since the initiation of the reform and opening-up policy more than four decades ago, China has registered substantial progress in grain production. Total grain output has grown consistently, rising from 305 billion kilograms in 1978 to over 1,392 billion kilograms in 2021. This expansion has largely enabled national self-sufficiency and, consequently, has strengthened food security. Over the same period, the average grain yield per unit area increased from 300 kg/mu in 1978 to 691 kg/mu in 2021, a rise that helped reduce regional productivity gaps and promote the scaling-up and industrialization of grain farming. The supporting industrial system has undergone continuous improvement, and the national capacity to safeguard grain quality and safety has been markedly enhanced. Through the establishment of quality and safety traceability systems alongside inspection and quarantine mechanisms, the incidence of mold contamination and residues of toxic and hazardous substances has been lowered, further reinforcing grain quality and safety assurance. In addition, the grain distribution system has been progressively refined. A market-oriented grain circulation structure, primarily driven by government procurement, has been instituted, effectively dismantling the regional barriers that previously hindered the movement of grain.

Following its accession to the WTO, China has become an active participant in global food security governance and has progressively constructed an open and orderly grain trade regime, substantially enhancing the openness of its grain economy. With the sustained expansion of grain trade, domestic and international grain markets have grown increasingly interconnected, and their price movements display a notable degree of synchronicity. Existing studies indicate that developing countries are particularly susceptible to the adverse consequences of international grain price volatility (5, 6). Such fluctuations not only affect domestic agricultural sectors but can also induce broader market instability, thereby threatening economic stability and sustainable growth. Consequently, within a rapidly transforming international trade landscape, stabilizing food prices and supply has become critically important for the sustainability of food consumption (7).

According to data from the General Administration of Customs, China’s grain import share has remained substantial in recent years. In 2021, for example, the country imported 164.539 million tonnes of grain, equivalent to 24.1% of its total domestic grain production; in 2022, imports totalled 146.872 million tonnes, accounting for 21.4% of total output. This high import dependence renders China susceptible to risks and spillovers from global grain markets, and sharp fluctuations in international food prices could have significant implications for China’s food security and, by extension, its national security. A review of China’s grain market development reveals that the market environment has been complex and volatile, with the industry and market subject to numerous uncertainties (8). Despite increased market openness, domestic grain prices remain only partially exposed to international price fluctuations. Since the early 21st century, geopolitical conflicts have evolved from episodic events into a systemic and recurrent risk, profoundly disrupting global food trade and pricing systems. According to data from the Food and Agriculture Organization (FAO), the FAO Cereal Price Index surged to a record high of 162.7 points in 2022, with the Russia-Ukraine conflict alone disrupting over 25 percent of global wheat exports. This elevated volatility directly endangers the food security of import-dependent nations. This empirical reality, however, presents a compelling theoretical tension. The Law of One Price in international trade theory posits that, under conditions of market openness, cross-border price arbitrage should lead to price convergence. In practice, China operates a hybrid institutional structure: tariff-rate quotas protect wheat and corn from full international price pass-through, whereas soybeans, with over 85 percent import dependence, are quota-free and fully exposed to global market forces. This institutional configuration of partial openness and partial regulation raises a critical question: how does this structure distort the transmission of price risk from international to domestic markets under geopolitical shocks? Understanding this distortion is particularly urgent given the systemic nature of contemporary geopolitical risks, which generate considerable uncertainty in the relationship between international and domestic grain prices beyond the short-term shocks captured by existing event studies.

While existing studies have examined price linkages, they often overlook the dynamic evolution of risk spillovers and the specific role of geopolitical factors in this process (9). To fill this gap, this study aims to accurately quantify the dynamic risk spillover between international and domestic grain markets and to empirically assess the impact of geopolitical uncertainties on it, thereby providing a scientific basis for guarding against externally transmitted risks and strengthening national food security. Building on this objective, the paper first applies the DCC-GARCH model to capture the time-varying characteristics of price correlations between international and domestic markets for different grain varieties. Second, the Generalized Forecast Error Variance Decomposition (GFEVD) approach is used to measure risk spillover effects within grain markets, allowing for a more detailed investigation of the risk linkages between international and domestic markets. Finally, the effect of geopolitical risk on total and net grain market spillovers is examined, after which a Time-Varying Parameter Vector Autoregression (TVP-VAR) model is employed to explore how the Geopolitical Risk Index influences grain market risk spillovers at different time periods and specific points in time.

This paper makes three key contributions to the extant literature. (1) We employ the DCC-GARCH model to characterize the dynamic evolution of price volatility spillovers between international and domestic grain markets, and subsequently apply the GFEVD framework to measure the magnitude of these spillovers. This integrated approach enables a more precise examination of the time varying interdependence between international and domestic grain price fluctuations, an issue that prior research based on event studies has been unable to address systematically. (2) While previous studies have largely focused on the effects of geopolitical risks on equity and oil market volatility or on price movements within a single commodity, they have generally neglected spillover effects across different grain varieties. By introducing geopolitical risk as an explanatory factor, we elucidate the dynamic volatility linkages among wheat, corn, and soybean markets, thereby offering a variety differentiated perspective that has been absent in existing work. (3) We systematically apply the TVP-VAR model to assess the impact of geopolitical risks on grain market risk spillovers from a dynamic and non-linear standpoint, and we also examine the effects of geopolitical risks during specific episodes on these spillovers. This allows our analysis to extend beyond short term episodic effects toward a dynamic understanding of how risk transmission evolves over time. Theoretically, we contribute to the existing literature by distinguishing the divergent effects of geopolitical threats (GPRT) and geopolitical actions (GPRA) at the risk spillover level. Our findings reveal that latent threats amplify volatility through expectation channels, whereas realized actions tend to stabilize markets via premeditated policy responses. This nuanced differentiation, enabled by our time-varying analytical framework, moves beyond the episodic treatment of geopolitical risk in prior event studies and captures its evolving, heterogeneous nature.

## 2 Literature review

Geopolitical risk is a pivotal concept in economics and international relations, and its prominence is closely tied to the 1970s oil crisis. During that period, heightened political tensions in the Middle East prompted the Organization of the Petroleum Exporting Countries (OPEC) to curtail oil supplies, which triggered a sharp rise in global oil prices (10). This episode illustrated the profound consequences that cross-border political events and decisions can have on the global economy, thereby stimulating scholarly and policy interest in geopolitical risk (11). Geopolitical risk is commonly understood as the uncertainty and associated risk that political events or decisions within a single country or region impose on the economic activities of other countries or regions (12). Such risks can originate from wars, coups, terrorism, sanctions, and other forms of political disruption, which are capable of impeding capital flows and the supply chains of goods and services, consequently affecting international trade and investment (13). With the acceleration of globalization and the growing intricacy of international relations in recent years, the influence of geopolitical risk on the global economy has intensified (14). For instance, political instability and conflicts in the Middle East, trade wars, and sanctions among major powers have substantially disrupted global supply chains and financial markets (15). In an effort to reduce these risks, many countries and corporations have adopted a variety of strategies, including supply chain diversification, financial hedging, and political risk insurance (16). Nonetheless, the inherent uncertainty and complexity of geopolitical risk render it especially difficult to predict and manage (17).

The spillover effects of price volatility have long constituted a central concern in economics, particularly within financial and commodity markets. Early studies focused predominantly on volatility relationships within a single market or between two markets, such as the linkages between equity and bond markets (18). These investigations commonly employed linear models, including co-integration analysis and vector autoregression models, to examine both long-run and short-run price relationships across different markets (19). Over time, the analytical focus expanded from single or paired markets to multiple interconnected ones, incorporating broader macroeconomic and international factors, such as global financial crises and monetary policy (20). This transition reflects the growing impact of globalization and financial market integration on price dynamics. In more recent years, alongside the increasing complexity of financial markets and the development of innovative financial instruments, research attention has shifted further toward the examination of price volatility relationships across multiple markets, asset classes, and time horizons, including interactions among equity, bond, foreign exchange, and commodity markets (21). Beyond the magnitude and direction of price volatility, these studies also emphasize its persistence, transmission mechanisms, and broader economic implications. Moreover, scholars have begun to explore the underlying causes and contributing factors of volatility spillovers, such as market participants’ behavior, information asymmetry, and market frictions (22).

A growing body of literature has documented the significant influence of geopolitical risk on both intra-market and inter-market volatility. Guidolin and La Ferrara (23) demonstrated that the outbreak of military conflicts elicits heterogeneous responses across stock, oil, and broader commodity markets. Employing an enhanced event study methodology, Ramiah et al. (24) investigated the impact of terrorist attacks on commodity markets, concluding that such events heighten market uncertainty and that equity markets tend to react more rapidly than commodity markets. Owing to the absence of continuous quantitative indicators for tracking geopolitical risk, existing studies have primarily relied on event study approaches to capture its effects. The literature examining the nexus between uncertainty and commodity markets generally explores how various forms of uncertainty relate to individual commodities. Balcilar et al. (25) adopted a non-parametric quantile technique to analyze the relationship between uncertainty and gold returns, while Joëts et al. (26) further examined the link between macroeconomic uncertainty and price volatility across different commodity classes. Geopolitical risk, a major source of uncertainty, is most frequently studied in relation to equity and oil markets (27, 28). Hu et al. (29) utilized the GARCH-MIDAS model to assess the effect of geopolitical uncertainty on the price volatility of crude oil, heating oil, and natural gas, but their analysis did not reveal the interconnections between geopolitical risk and these three commodity markets. Liu et al. (30) applied the framework of Diebold and Yilmaz (31) to explore how macroeconomic, capital market, and geopolitical risks drive the price volatility of soybeans, gold, and crude oil.

Beyond the general literature on price transmission and geopolitical risk, a growing body of research has specifically examined how China’s distinctive grain policy institutions shape the international-domestic price linkage. Two institutional features are particularly relevant. First, the tariff-rate quota (TRQ) system for wheat and corn, which permits low-tariff imports only up to a designated quota volume and imposes substantially higher out-of-quota tariffs, has been demonstrated to create a “price band” within which domestic prices can fluctuate without being fully driven by international benchmarks (30, 32). Empirical studies have documented that the insulation effect is strongest for wheat, moderate for corn, and virtually absent for soybeans, which are quota-free (33, 34). Second, the minimum purchase price (MPP) policy for wheat and rice establishes government-administered price floors that dominate domestic market expectations, effectively weakening the transmission of international price declines while allowing international rises to pass through more readily (35–37). This asymmetric transmission pattern has been identified as a key feature of China’s grain market integration. Moreover, the 2016 abolition of the temporary corn reserve policy in Northeast China, which replaced state-administered procurement with a “market-based procurement plus subsidy” system, constitutes a pivotal institutional breakpoint. Recent studies have shown that this reform abruptly increased the co-movement between domestic and international corn prices, fundamentally altering the price transmission structure that had prevailed since the early 2000s (38). These institutional studies provide important context for interpreting the time-varying correlation patterns observed in our empirical analysis and underscore the need to distinguish between long-term structural changes and short-term geopolitical impulses in explaining grain price dynamics.

While the existing literature has substantially advanced our understanding of the interplay between geopolitical risk and commodity markets, several critical gaps remain underexplored. (1) Most studies have concentrated on crude oil and equity markets (39, 40), with agricultural commodities, particularly grain markets, receiving disproportionately limited attention. When grain markets are examined, the analysis tends to be restricted to a single crop or a single market, largely neglecting the cross market spillover dynamics that are essential for understanding systemic risk transmission. (2) Existing studies have predominantly relied on event study approaches that capture short term, episode specific effects of geopolitical events (41–45). While valuable for identifying immediate market reactions, these approaches are inherently limited in capturing the time varying, dynamic nature of risk transmission that unfolds over extended periods and across different market conditions. The continuous evolution of geopolitical risks, from latent threats to realized actions, necessitates a dynamic analytical framework that can track spillovers as they evolve. And (3) most importantly for our purposes, there is a conspicuous absence of systematic, variety based spillover analysis that distinguishes how different grain types respond to geopolitical shocks (46, 47). Wheat, corn, and soybeans hold fundamentally distinct roles in China’s food security architecture: wheat as a core staple protected by strategic reserves, corn as a dual purpose feed and food crop with growing import elasticity, and soybeans as a highly import dependent commodity exposed to global supply chain disruptions. However, existing studies rarely disaggregate these heterogeneous responses, instead treating grain markets as homogeneous entities. Such aggregation masks the distinct transmission mechanisms and policy implications associated with each crop.

This study aims to address these gaps by providing a systematic, dynamic, and variety differentiated analysis of geopolitical risk spillovers in grain markets. Specifically, we contribute to the literature by: (1) capturing time varying correlations between domestic and international prices using the DCC GARCH model, (2) quantifying the magnitude and direction of risk spillovers across multiple grain varieties through the GFEVD framework, and (3) assessing the dynamic impact of geopolitical risk on these spillovers with the TVP VAR approach. By explicitly disaggregating wheat, corn, and soybeans, we provide a nuanced understanding of how different staple crops respond to geopolitical uncertainties, offering both theoretical insights and practical guidance for food security policy in importing countries like China.

To ground our empirical analysis in a coherent theoretical framework, we delineate the conceptual transmission channels through which geopolitical risk influences grain market spillovers. Geopolitical risk operates through three primary channels. The trade policy channel emerges when geopolitical tensions induce tariffs, export bans, or sanctions that directly constrain trade flows and alter price expectations. The logistics disruption channel materializes when military conflicts damage agricultural infrastructure or blockade shipping routes, thereby physically restricting supply. The cost push channel transmits geopolitical shocks indirectly via elevated freight rates, exchange rate volatility, and surging energy and fertilizer prices, all of which raise global production costs.

Within this framework, crop specific heterogeneity in spillover responses is theoretically determined by three structural factors. First, trade policy protection: crops such as wheat, which are shielded by import quotas and strategic reserves, act as net transmitters of policy driven volatility when geopolitical tensions prompt stockpiling behavior. Second, import dependence: crops such as soybeans, with over 85 percent reliance on foreign sources, are inherently exposed to external shocks but function as net receivers, because increased import costs are absorbed by domestic processors rather than transmitted to other crops. Third, cross crop substitution elasticity: corn and soybean meal serve as substitutes in animal feed, generating bidirectional pairwise spillovers through feed formula adjustments when relative prices change.

This framework yields three testable predictions: (i) geopolitical shocks reduce total connectedness as markets fragment; (ii) wheat exhibits positive net spillovers under geopolitical stress; and (iii) soybeans exhibit negative net spillovers, while corn-soybean pairwise spillovers remain elevated. These predictions guide the empirical analysis that follows.

## 3 Model specification and data description

### 3.1 Model specification

To achieve the research objectives, we will conduct quantitative analysis using dynamic conditional correlation generalized autoregressive conditional heteroskedasticity (DCC-GARCH) and the Generalized Forecast Error Variance Decomposition (GFEVD). DCC-GARCH is employed to characterize the time varying conditional correlations between domestic and international grain prices. The model examines and answers whether and how price co-movements change over time. It can accurately capture dynamic correlation dependencies across markets without requiring any pre-specified fixed relationships. GFEVD is used to measure the magnitude, direction, and intensity of risk spillovers among grain markets. While DCC-GARCH reveals the existence and evolution of correlations, GFEVD further decomposes the forecast error variance of each market to identify which markets are net transmitters of price shocks and which are net receivers. GFEVD addresses the questions of where risks are transmitted and from whom to whom. Finally, we will employ the TVP-VAR model to explore how geopolitical risks drive the observed spillover patterns, thereby shifting the relationship between variables from correlation measurement to causal analysis.

#### 3.1.1 DCC-GARCH model

The DCC-GARCH model, proposed by Engle (48), extends the traditional GARCH model to capture the dynamic conditional correlations in financial time series.

Assume the domestic wheat price return is *r*_1t_ and the foreign wheat price return is *r*_2t_. The DCC-GARCH model can be expressed as follows:


$$\begin{array}{c} r_{i\,t}=\mu_{i}+{\text{Ò}}_{i\,t}\\ {\text{Ò}}_{i\,t}=h_{i\,t}\,z_{i\,t}\\ h_{i\,t}^{2}=\omega_{i}+\alpha_{i}\,{\text{Ò}}_{i,t-1}^{2}+\beta_{i}\,h_{i,t-1}^{2} \end{array}$$ (1)

In Equation 1, h_*it*_ represents the conditional volatility of wheat i at time t, and z_*it*_ denotes an independently and identically distributed random error term. The dynamic conditional correlation can be expressed as Equation 2:


$$q_{i\,t,j}=\left(1-a-b\right)\,\bar{q}+a\,\epsilon_{j,t-1}+b\,q_{i\,j,t-1}$$ (2)

$$p_{i\,j,t}=\frac{q_{i\,j,t}}{\sqrt{q_{j,t}}}$$ (3)

In Equation 3, ρ_*ij*,*t*_ represents the conditional correlation between wheat *i* and *j* at time *t*, *q*_*ij,t*_ denotes the conditional covariance between wheat *i* and *j* at time *t*, and *a* and *b* are the model parameters. The term $\bar{q}$ signifies the unconditional mean of the covariance.

#### 3.1.2 GFEVD model

The generalized forecast error variance decomposition (GFEVD), introduced by Diebold and Yilmaz (31), offers a multivariate time-series framework that quantifies the relative contribution of each variable to the forecast error variance of all other variables within the system. In contrast to the traditional forecast error variance decomposition, the GFEVD is invariant to the ordering of variables and simultaneously reveals both the direction and intensity of risk spillovers across markets.

Based on this, we construct a grain market risk spillover index model. A simplified VAR(p) model is set as follows:


$$X_{t}=A_{1}\,X_{t-1}+A_{2}\,X_{t-2}+\ldots +A_{p}\,X_{t-p}+u_{t}$$ (4)

In Equation 4, *X*_*t*_ is a *k*×1 vector containing the price returns of domestic and international wheat, corn, and soybeans. *A*_1_,*A*_2_,…,*A*_*p*_ is a *k* × *k* parameter matrix, and μ_*t*_ is a *k*×1 error term vector that follows a normal distribution with a mean of 0 and a covariance matrix Σ.

To calculate the GFEVD, the VAR(p) model is first transformed into its *MA*(∞) representation:


$$X_{t}=\sum_{i=0}^{\infty }\Psi_{i}\,u_{t-i}$$ (5)

In Equation 5, Ψ_0_=*I*_*k*_ is a *k* × *k* identity matrix, and Ψ_*i*_ can be recursively calculated using the VAR coefficients. In this representation, the current value of each variable can be viewed as a function of past shocks.

For a specific shock j and the forecast error variance of variable i, the GFEVD can be expressed as:


$$G\,F\,E\,V\,D_{i,j}\,\left(h\right)=\frac{\sum_{t=0}^{h-1}{\left[\Psi_{t}\,\Sigma \,e_{j}\right]}^{2}}{\sum_{t=0}^{h-1}{\left[\Psi_{t}\,\Sigma \,e_{i}\right]}^{2}}$$ (6)

In Equation 6, Σ is the covariance matrix of the error terms, and *e_i_*is a selection vector with one on the i-th element and zeros elsewhere. This represents the contribution of shock *j* to the forecast error variance of variable *i* over the forecast horizon *h*.

Applying this model to the price returns of wheat, soybeans, and corn allows us to measure how price shocks in domestic and international markets affect the price volatility in other markets, providing detailed insights into price transmission and shock spillovers.

#### 3.1.3 TVP-VAR model

This paper further employs the Time-Varying Parameter Vector Autoregression (TVP-VAR) model to analyze the dynamic relationship between geopolitical risk and grain risk spillovers. The TVP-VAR model is specified as follows:


$$Y_{t}=X_{t}\beta_{t}+M_{t}^{-1}\sum {}_{t}$$ (7)

In Equation 7, Y_*t*_ represents the grain risk spillover index, X_*t*_ denotes the geopolitical uncertainty index, β_*t*_ is the time-varying autoregressive coefficient, and M_*t*_ contains the elements *m*_*t*_ = (*m*_1*t*_, *m*_2*t*_, … ,*m*_*kt*_) ^′^ in its lower triangular form. Assuming $\sigma_{j\,t}^{2}=\beta \,e\,x\,p\,\left(h_{j\,t}\right)$ is the stochastic volatility and *h*_*t*_ = (*h*_1*t*_, *h*_2*t*_, … ,*h*_*kt*_)^′^ is the random fluctuation, the parameters in Equation 5 follow a random walk process defined as:


$$\begin{array}{c} \beta_{t+1}=\beta_{t}+\mu_{\beta_{t}}\\ m_{t+1}=m_{t}+\mu_{a_{t}}\\ h_{t+1}=h_{t}+\mu_{h_{t}} \end{array}$$ (8)

$$\left[\begin{array}{c} \varepsilon_{t}\\ \mu_{\beta_{t}}\\ \mu_{m_{t}}\\ \mu_{h_{t}} \end{array}\right]∼\left[0,\left[\begin{array}{c} I\\ 0\\ 0\\ 0 \end{array}\,\begin{array}{c} 0\\ \sum_{\beta }\\ 0\\ 0 \end{array}\,\begin{array}{c} 0\\ 0\\ \sum_{m}\\ 0 \end{array}\,\begin{array}{c} 0\\ 0\\ 0\\ \sum_{h} \end{array}\right]\right]$$ (9)

In Equations 8, 9, $\beta_{\beta +1}∼N\left(\mu_{\beta_{0}},\sum_{\beta_{0}}\right),m_{s+1}∼N(\mu_{m_{0}},$$\sum_{m_{0}}),$*h*_*s* + 1_∼*N*$\left(\mu_{h_{0}},\sum_{h_{0}}\right)$. The Monte Carlo method is employed to perform simulation sampling and obtain the posterior distribution of the model parameters.

### 3.2 Data sources and processing

To study the relationship between geopolitical risk and domestic and international grain markets, this paper focuses on the prices of major grains, such as wheat, soybeans, and corn, along with geopolitical risk indicators.

(1) Major grain prices: Among the principal agricultural commodities in China, wheat, soybeans, and corn each hold a central position, though their roles and trade profiles differ substantially. Among these, corn has the largest sown area, wheat is a fundamental staple, and soybeans represent a key economic crop, each with distinct characteristics and economic importance. The extent of their involvement in international trade varies markedly. China applies import quotas to wheat and corn, a measure that restricts the share of these two grains in global trade. By contrast, soybeans are characterized by exceptionally high import dependency, with imports accounting for more than 85 percent of domestic consumption.

International prices for U.S. No. 1 Hard Red Winter Wheat, U.S. No. 2 Yellow Corn, and U.S. Soybeans (in USD/metric ton) were sourced from the IMF’s primary commodity price database. Domestic prices for wheat, corn, and soybeans were obtained from the “China Agricultural Product Price Survey Yearbook.” Due to differences in pricing units, domestic prices were converted to “yuan per ton” using the relevant exchange rates for consistency. The sample period spans from January 2003 to May 2023. Considering the potential impact of seasonal factors on monthly frequency data, the Census X12 method was used for seasonal adjustment. To ensure data stationarity, price returns were used to reflect grain price changes, calculated as $R_{t}=100\times \ln\left(\frac{P_{t}}{P_{t-1}}\right)$, where *R*_*t*_ is the price return at time *t*, and *P*_*t*_ is the grain price at time *t*. After processing, the price returns for domestic and international wheat, corn, and soybeans were obtained and denoted as WWHEAT, WCORN, WSOYBEANS for international returns, and CWHEAT, CCORN, CSOYBEANS for domestic returns.

**FIGURE 1:**
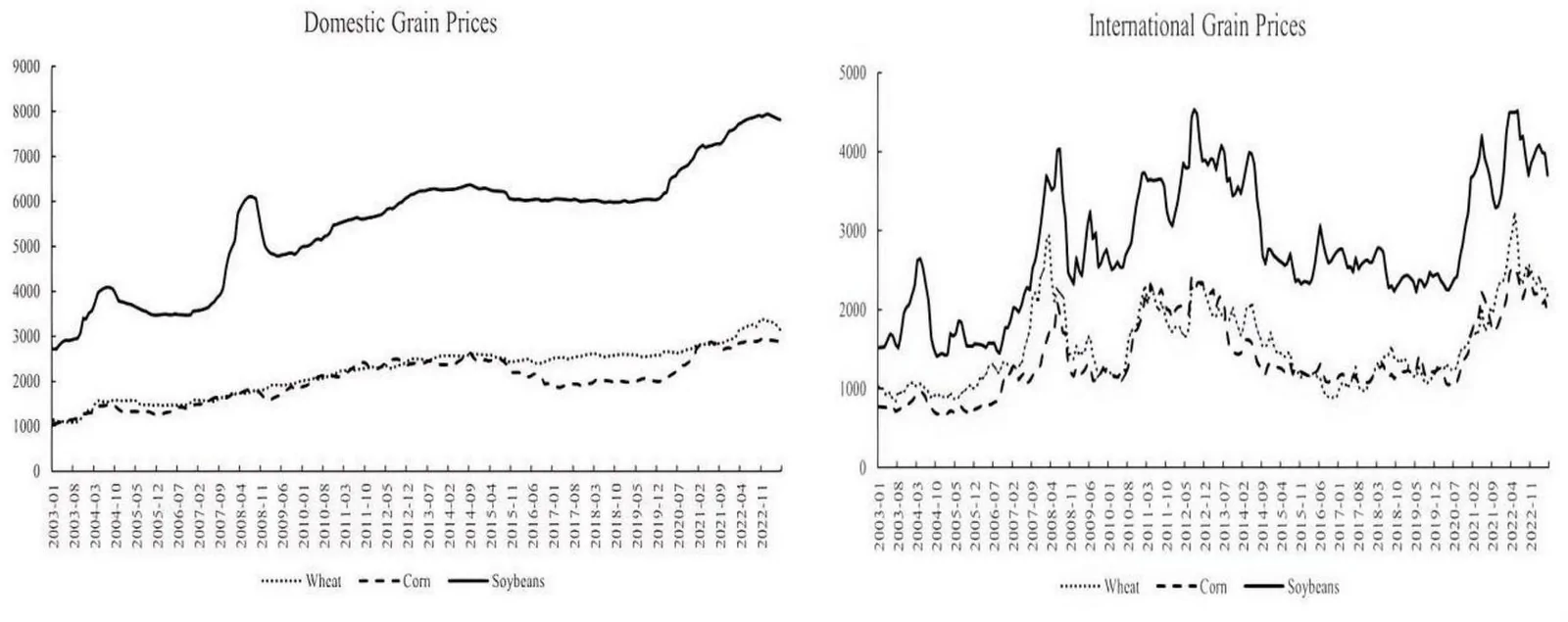
Trends in domestic and international grain prices.

Figure 1 shows that since 2003, both domestic and international grain prices have exhibited an upward trend. The increase in domestic soybean prices was particularly significant between 2003 and 2008, after which it gradually stabilized. Overall, international grain prices have fluctuated more frequently, while domestic prices have remained relatively stable.

(2) This study adopts the Geopolitical Risk (GPR) Index developed by Caldara and Iacoviello (49) to measure geopolitical risk. The GPR Index is designed to capture risks arising from wars, terrorist activities, and political tensions that have the potential to disrupt the stability and peace of international relations. To construct the index, articles addressing geopolitical risk are identified and counted from 11 major international newspapers, including the Boston Globe, Chicago Tribune, Financial Times, New York Times, and Wall Street Journal. The monthly volume of such articles is then expressed as a proportion of the total number of articles published that month, and the resulting ratio is standardized to a mean value of 100.

To enhance precision in measurement, six keyword categories were established. The first category captures geopolitical risks and military tensions involving the United States or occurring on a global scale. The second category focuses on nuclear tensions. The third and fourth categories address threats of war and terrorism, respectively. The fifth and sixth categories relate to actual geopolitical events, such as terrorist attacks and the outbreak of armed conflicts. Building on this keyword structure, Caldara and Iacoviello (49) further introduced two sub-indices: the Geopolitical Threats (GPRT) Index and the Geopolitical Acts (GPRA) Index. The GPRT Index incorporates keywords from the first four categories, whereas the GPRA Index draws on the fifth and sixth categories, thus enabling a more precise distinction between geopolitical events and the risks associated with them.

This study primarily uses the global and China-specific geopolitical risk index, the geopolitical threats (GPRT) index, and the geopolitical acts (GPRA) index. Seasonal adjustments to the geopolitical risk indices were made using the Census X12 method. Data were sourced from the Economic Policy Uncertainty Database.^1^

**FIGURE 2:**
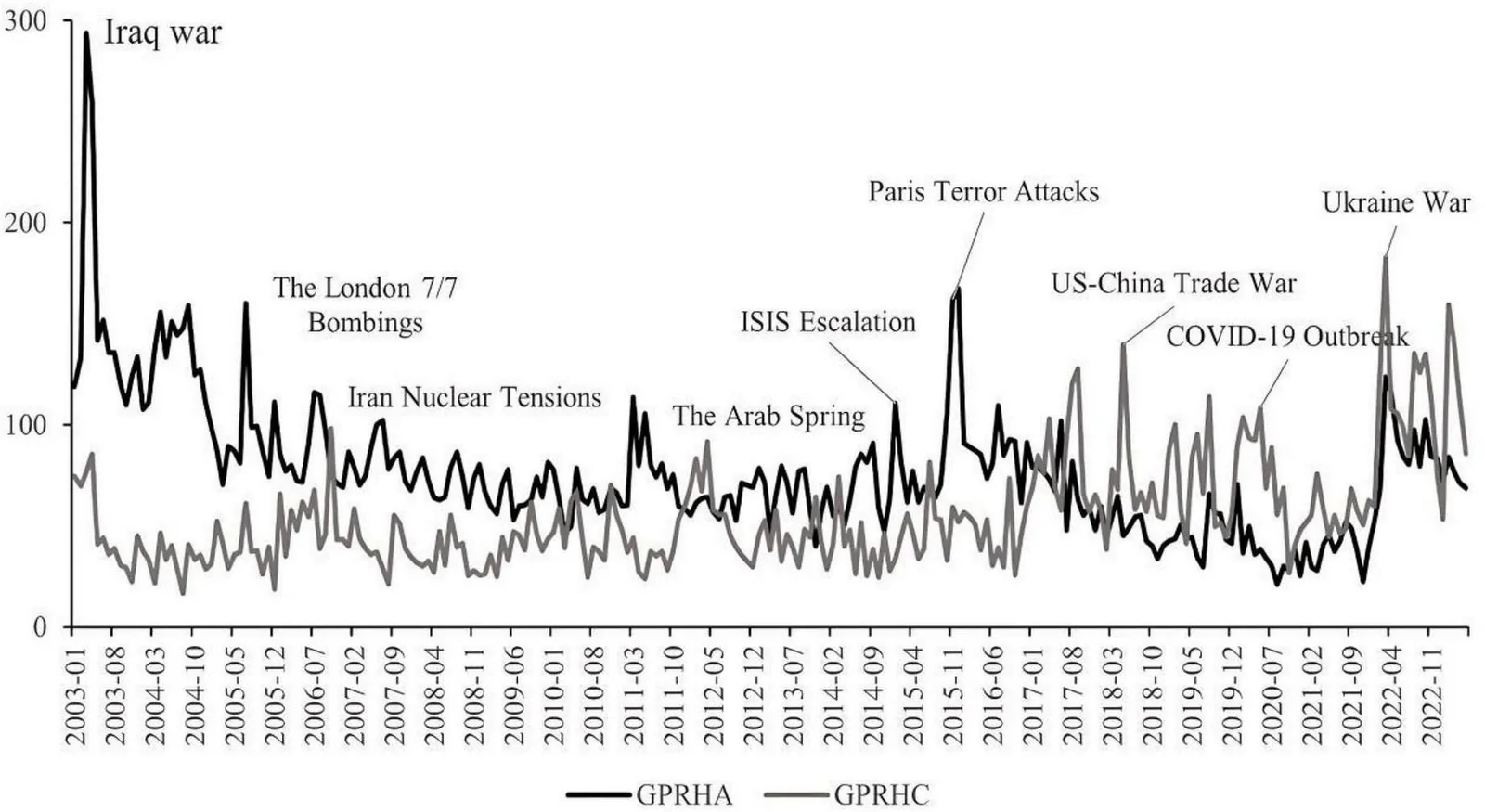
Trends in global and China’s geopolitical risk index.

Figure 2 presents the temporal evolution of the global geopolitical risk index and the China-specific geopolitical risk index over the period 2003–2022. In this figure, GPRHA is employed to represent the global index, while GPRHC is used to represent the China–specific index. As shown, both series exhibit pronounced volatility, reflecting the fluctuating intensity of international tensions and geopolitical conflicts throughout the two decades. The Geopolitical Risk Index displayed pronounced fluctuations over the period from 2003 to 2022, reflecting both global and domestic economic risks, including those transmitted to grain markets. In April 2003, the Iraq War triggered significant volatility in global oil prices, which in turn undermined domestic economic stability. The 2011 Syrian conflict drove a further increase in the index, and political unrest in Egypt produced another peak in September 2013. Russia’s annexation of Crimea in March 2014 precipitated the Ukraine crisis, resulting in a moderate peak that same month. Geopolitical risk escalated once more in September 2014 as tensions between the United States and Russia intensified and economic sanctions were imposed on Russia, further amplifying market uncertainty.

Toward the end of 2015, France experienced a series of terrorist attacks. From 2016 onward, geopolitical risk volatility intensified, generating several moderate peaks. Notable peaks emerged in August 2017, driven by the Barcelona terrorist attack and unrest in India; in May 2018, coinciding with the US withdrawal from the Iran nuclear deal, US airstrikes in Syria, escalating Middle East conflicts, and attacks in Libya; and in August 2019, corresponding to conflict in Saudi Arabia and the expiration of the Intermediate-Range Nuclear Forces (INF) Treaty. The COVID-19 pandemic subsequently propelled the index to its highest point since 2008. In February 2022, Russia’s full-scale invasion of Ukraine constituted a landmark event, representing the most consequential development since the outbreak of the war in Ukraine in 2014.

## 4 Empirical results

### 4.1 Dynamic correlation analysis

This study utilizes the DCC-GARCH model to analyze the dynamic correlation between domestic and international grain markets. Prior to model estimation, ARCH effect tests were conducted on the domestic and international grain price variables, revealing significant ARCH effects for all grain price variables.

**FIGURE 3:**
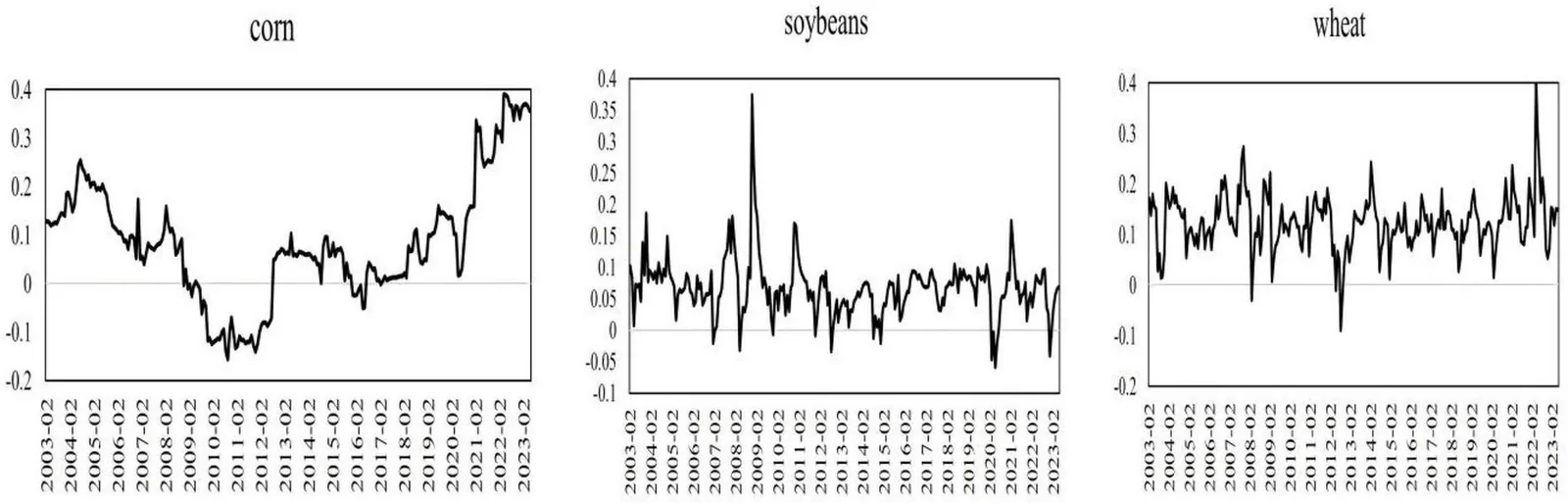
Dynamic correlation between international and domestic grain prices.

Figure 3 presents the dynamic correlation coefficients between domestic and international prices for different grain varieties. First, the correlation between domestic and international corn prices is the strongest, followed by that of wheat, while soybeans exhibit the weakest correlation. Second, these dynamic correlation coefficients display pronounced time-varying behavior, with corn prices showing the greatest variability in their correlation levels. Third, during the global food crisis from 2008 to 2010, the correlation between domestic and international soybean prices experienced substantial fluctuations. Similarly, wheat prices demonstrated considerable fluctuation during the Russia-Ukraine war in March and April 2022, with corn prices exhibiting a similar pattern to a lesser degree. These observations suggest that geopolitical risks exert a significant influence on the dynamic correlation between international and domestic grain price volatility. However, a more nuanced interpretation of corn price dynamics is warranted. The V-shaped pattern in corn’s dynamic correlation, declining before 2016 and rebounding sharply thereafter, is not primarily driven by geopolitical forces but rather by a fundamental institutional shift in China’s domestic grain policy. In 2016, the Chinese government abolished the temporary corn reserve policy in Northeast China and replaced it with a “market-based procurement plus subsidy” system. Prior to this reform, state-led procurement at administratively set prices had artificially insulated domestic corn prices from international market signals. The post-2016 liberalization exposed domestic corn prices to global market forces, abruptly strengthening the co-movement between international and domestic prices. This policy rupture constitutes the primary driver of the V-shaped reversal observed in our DCC-GARCH estimates. Geopolitical risks, including the Russia-Ukraine conflict in 2022, merely superimposed short-term impulses onto this longer-term structural trend, amplifying volatility within a market that had already become more integrated with global prices. For wheat and rice, by contrast, the continued operation of the Minimum Purchase Price policy has maintained a degree of insulation, explaining their comparatively lower and more stable correlation coefficients.

International food price volatility can be primarily attributed to several interrelated factors. First, a persistent mismatch between grain supply and demand has exerted strong upward pressure on prices. On the supply side, frequent extreme weather events in major crop-producing countries have led to low inventory levels and tightened supply conditions. Since 2020, severe droughts in Brazil have exacerbated production shortfalls in sugarcane, soybeans, corn, and coffee, thereby disrupting global supply chains. At the same time, natural disasters in the United States and Europe have further reinforced expectations of reduced global grain output and depleted stocks. In April 2022, the U.S. Department of Agriculture reported in its Crop Progress assessment that 2021/22 production forecasts for corn in Brazil and Argentina were revised downward by 3.5 million tonnes and for soybeans by 25 million tonnes, while the planting progress for soybeans and wheat in the United States lagged behind the previous year’s levels by 22% and 48%, respectively.

On the demand side, robust global appetite for grains has contributed to rising prices. Current strong market demand, coupled with increased precautionary stockpiling, has created a substantial supply-demand gap. According to estimates by the Food and Agriculture Organization, the world population is projected to reach 10 billion by 2050, implying a 50% increase in food demand. Moreover, China’s soybean imports have consistently remained in the range of 80–90 million tonnes in recent years, accounting for more than 80% of its total soybean consumption, and these import volumes are expected to continue their upward trajectory.

The pronounced heterogeneity in dynamic correlations across grains can be attributed to their distinct trade regimes and downstream demand structures. The highest correlation observed for corn reflects its dual role as both a staple food and a primary feed ingredient in China. The expansion of the domestic livestock sector has created persistent and elastic import demand, making domestic corn prices highly sensitive to international benchmark prices, as global price signals are quickly transmitted through feed import channels. In contrast, the weakest correlation for soybeans is counterintuitive given China’s over-85% import dependence. However, this can be explained by two factors. First, China’s soybean imports are largely conducted via long-term futures contracts with a 2- to 3-month shipping lag, which temporarily decouples domestic spot prices from immediate international spot fluctuations. Second, international soybean prices are heavily influenced by financial speculation on the Chicago Board of Trade (CBOT) and weather conditions in the Americas, whereas domestic prices are also shaped by state reserve releases and crushing plant inventory cycles (50, 51). These financial and logistical frictions dampen the short-term pass-through of international price shocks, resulting in a lower time-varying correlation.

### 4.2 Spillover effects in the grain market

#### 4.2.1 Static spillover effects analysis

All grain price return series were first examined using the Augmented Dickey–Fuller (ADF) unit root test. The results confirm that each series is stationary at the 1% significance level, which provides a robust empirical basis for specifying the VAR model. Both the Akaike Information Criterion (AIC) and the Bayesian Information Criterion (BIC) identify an optimal lag length of one. Building on this specification, the generalized forecast error variance decomposition (GFEVD) framework is employed to examine the volatility spillover linkages between domestic and international grain markets, with detailed results reported in Table 1.

**TABLE 1:** Static spillover effects analysis.

Variables	CCORN	CSOYBEANS	CWHEAT	WCORN	WSOYBEANS	WWHEAT	FROM
CCORN	55.26	14.93	17.13	3.32	6.85	2.5	44.74
	[45.02]	[9.43]	[12.13]	[2.01]	[3.92]	[1.36]	[28.84]
CSOYBEANS	7.45	61.93	5.03	6.3	13.07	6.23	38.07
	[6.33]	[46.66]	[3.68]	[3.9]	[8.29]	[3.71]	[25.91]
CWHEAT	14.42	8.85	66.23	1.69	6.06	2.75	33.77
	[11.96]	[5.84]	[52.91]	[1.12]	[3.88]	[1.66]	[24.47]
WCORN	0.32	2.68	0.1	54.43	24.86	17.61	45.57
	[0.28]	[1.93]	[0.09]	[47]	[20.79]	[14.24]	[37.34]
WSOYBEANS	0.57	2.05	0.19	25.28	54.51	17.4	45.49
	[0.55]	[1.73]	[0.17]	[21.03]	[46.24]	[13.98]	[37.46]
WWHEAT	0.51	1.61	0.37	17.88	17.08	62.54	37.46
	[0.48]	[1.42]	[0.3]	[15.98]	[15.36]	[55.21]	[33.54]
TO	23.26	30.12	22.82	54.48	67.93	46.49	245.11
	[19.59]	[20.35]	[16.37]	[44.04]	[52.24]	[34.96]	[187.55]
Net	−21.48	−7.95	−10.95	8.91	22.44	9.03	49.02/40.85
	[−9.25]	[−5.56]	[−8.1]	[6.7]	[14.78]	[1.41]	[37.51/31.26]

The aggregate risk spillover index for the grain market reaches 245.11%, pointing to close interconnectedness and pronounced spillover effects between domestic and international grain markets. This result emphasizes that grain market risks should be evaluated through a combined lens encompassing both domestic and international dimensions rather than by focusing on any single market. Within-market risk spillovers generally exceed cross-market spillovers, a pattern particularly visible in the domestic wheat market (CWHEAT) and the international wheat market (WWHEAT), which record own-market spillovers of 66.23% and 62.54%, respectively, followed by the domestic soybean market (CSOYBEANS).

The directionality of risk spillovers in the grain market is notably asymmetric. Outward spillover from the domestic market amounts to 116.58%, whereas that from the international market reaches 128.52%, indicating that the overall grain market system is more vulnerable to risk transmission emanating from international markets. The international soybean market (WSOYBEANS) reports a net risk spillover of 22.44%, which is higher than the net spillovers observed in the international corn market (WCORN, 8.91%) and the international wheat market (WWHEAT, 9.03%). The values shown in brackets in Table 1 reflect short-term estimates for the sample. Given the long-run linkages between international soybean and corn markets, the network may absorb this information more gradually, while the international wheat market displays greater short-term activity, evidenced by a volatility spillover of 7.62%.

#### 4.2.2 Dynamic spillover effects analysis

**FIGURE 4:**
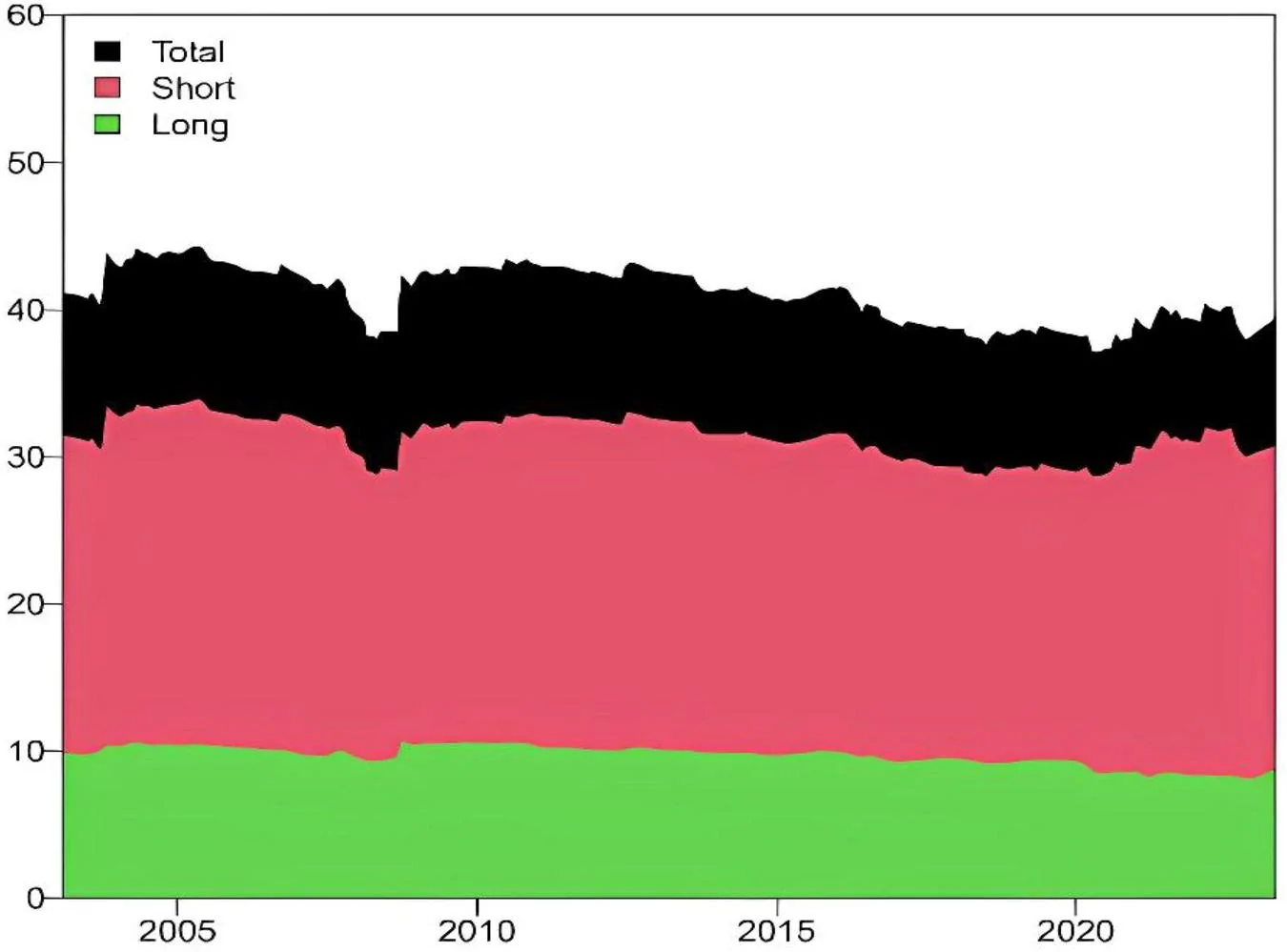
Dynamic changes in total risk spillover index in the grain market.

Static analysis offers an aggregate representation of the relationships among grain markets under equilibrium conditions, yet it remains insufficient for capturing the temporal dynamics of these interactions. To investigate the time-varying characteristics of risk spillover effects between domestic and international grain markets, a rolling-window approach was adopted, with a window length of 200 days and a step size of 10 days. In Figure 4, the black area depicts the dynamic total spillover index, while the green and red areas correspond to long-term low-frequency and short-term high-frequency spillovers, respectively. An increase in the total connectedness index (TCI) indicates more synchronized price movements across grain varieties and a higher likelihood of risk contagion within the network.

As shown by the black shaded area in Figure 4, the TCI exhibits a pronounced event-driven pattern, fluctuating mainly between 36.95% and 44.17%. At the beginning of 2003, the TCI stood at 41.08% and subsequently climbed to a peak of 43.68% in November of that year. Throughout 2004 and 2005, the index remained within the 42% to 44% band, reaching its highest value of 44.17% in May 2005. Thereafter, under the global influence of the 2007–2008 financial crisis and the European debt crisis, the TCI experienced a gradual decline, touching a low of 37.87% in March 2008. The Arab Spring in 2010 further intensified market instability, contributing to a progressive decrease in the index from 2011 through 2013, which settled at 41.60% in September 2013.

In 2020, the COVID-19 pandemic drove the index down to a trough of 37.11% in April, after which it gradually recovered. By 2021, the index had risen above 40%, reaching a peak of 40.08% in June. Throughout 2022, it fluctuated between 39% and 40%, declining moderately toward the end of the year, before rebounding to 39.39% in May 2023. Overall, the total connectedness index for domestic and international grain markets displayed alternating upward and downward movements, capturing the contagion dynamics and the overall degree of interconnectedness between these markets.

While the index’s evolution can be linked to the events above, a closer inspection of Figure 4 suggests that connectedness within this network is predominantly short-term in nature. When the high-frequency band governs system connectedness, it signals that the markets involved process information relatively quickly, so that shocks materialize mainly in the short run. This further implies that the reverberations of past shocks are not strong enough to outweigh the impact of current developments in individual markets on overall connectedness. In contrast, when the low-frequency band determines network connectedness, it more likely reflects structural changes that took place at some earlier point but became evident only gradually. With respect to the integration of domestic and international grain markets, the variables in the network are more tightly integrated over the short term, largely under the influence of global developments, and less integrated over the long term, owing to the absence of developments that fundamentally reshape market structures.

#### 4.2.3 Net spillover effects analysis

**FIGURE 5:**
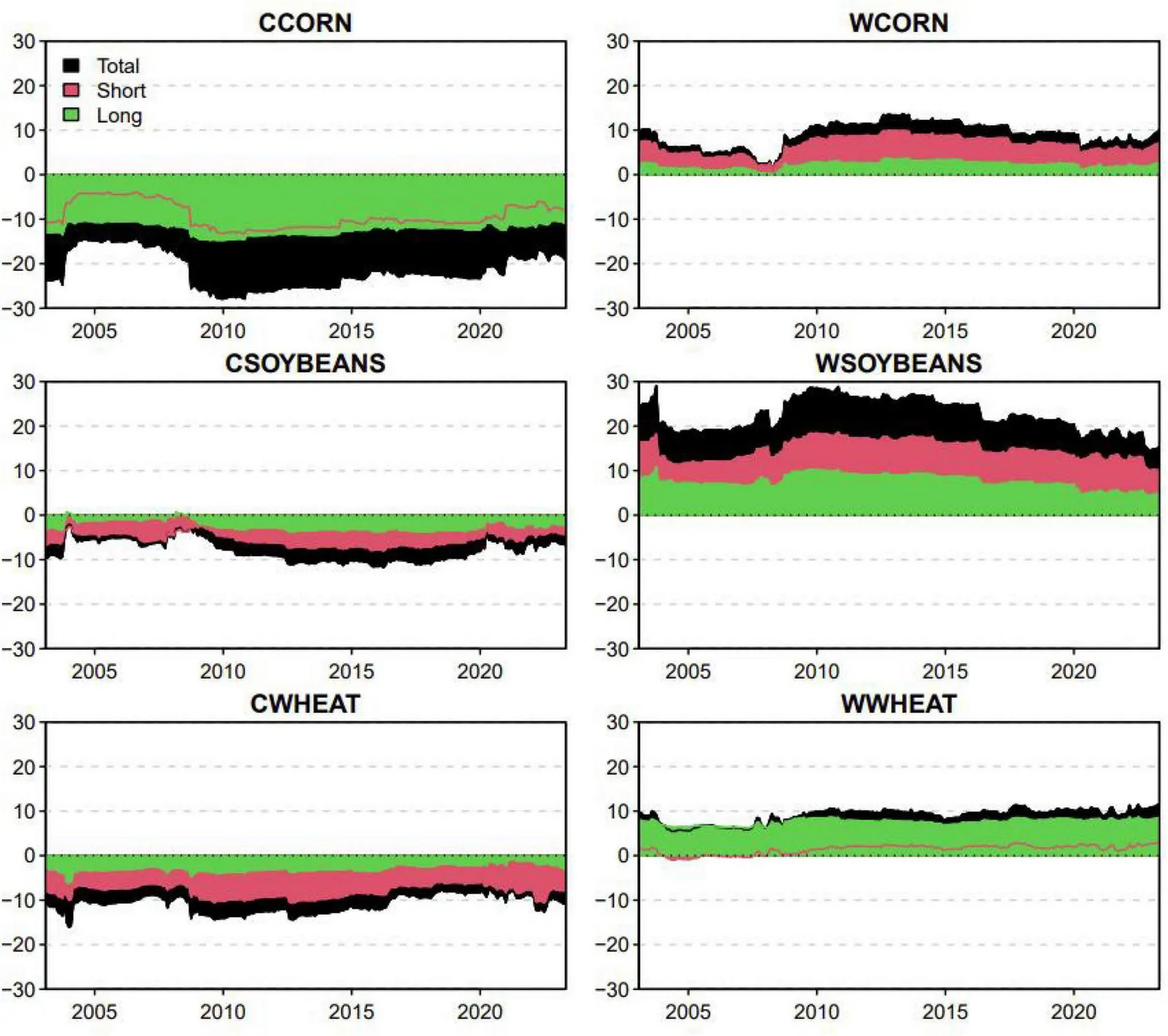
Net spillover direction in the grain market.

To distinguish the net contributors to and net recipients of risk within the network, this study computes net volatility spillover indices for both domestic and international grain markets. Figure 5 displays these indices, with the black, red, and green shaded regions representing overall, short-term, and long-term volatility spillovers, respectively. A positive value indicates that the corresponding grain variety functions as a net transmitter of price shocks to other grains in the system, whereas a negative value signals that it acts as a net receiver.

As depicted in Figure 5, the domestic grain market consistently acted as a net recipient of price disturbances throughout the sample period. Within this domestic segment, the connectedness of soybeans and wheat is predominantly short-run in nature, whereas the domestic corn market exhibits long-run connectedness. This pattern suggests that China’s substantial corn imports render the domestic corn market directly sensitive to external price movements, producing sustained co-movement. By contrast, domestic soybean and wheat markets are shaped more by short-term supply and demand fluctuations, policy adjustments, and other transitory influences. International grain markets serve as net transmitters of price shocks over the long term, reinforcing their central role in global grain supply chains. With respect to the horizon of shock transmission, international corn and soybeans are principally driven by short-run connectedness, while the international wheat market is dominated by persistent long-run shocks.

#### 4.2.4 Analysis of net pairwise spillover effects

The examination of net pairwise directional connectedness (NPDC) uncovers substantial spillover effects between domestic and international grain markets, with NPDC values generally ranging from 5% to 10%. Notably, the NPDC measures between the domestic corn and domestic soybean markets, as well as between the domestic and international soybean markets, exceed 10%. In contrast, NPDC values for the pairs involving international corn and international soybeans, domestic wheat and international corn, international corn and international wheat, and international soybeans and international wheat all remain below 5%. Around 2015, domestic soybeans were primarily influenced by international soybeans and wheat, while also transmitting shocks to domestic wheat. At the same time, domestic corn functioned as a major net receiver of price disturbances, driven chiefly by spillovers from domestic soybeans.

The substantial net pairwise spillover between domestic corn and soybeans, exceeding 10%, highlights the profound feed market substitution effect in China. Corn and soybean meal are the two main components of compound animal feed. When geopolitical events or weather shocks drive up corn prices, domestic livestock farmers tend to substitute corn with cheaper alternatives such as imported barley or reduce soybean meal inclusion rates, thereby transmitting price pressure between these two related markets. This substitution elasticity explains why the domestic corn-soybean nexus acts as a major conduit for internal risk transmission, a stylized fact that underscores the need for integrated policy management across feed grain inventories.

### 4.3 Analysis of shock effects

#### 4.3.1 Preliminary results of geopolitical risk impact

While earlier work has documented the spillover effects of risks within grain markets, the transmission mechanisms underlying these risks have yet to be systematically examined. This section investigates the impact of China’s geopolitical risk on the total and net spillovers in both domestic and international grain markets.

Table 2 presents the estimated effects of the China Geopolitical Uncertainty Index on the Total Connectedness Index (TCI) and the net spillovers for individual grain categories. The TCI coefficients are consistently negative and statistically significant across all specifications, indicating that heightened geopolitical uncertainty attenuates aggregate risk spillovers within grain markets. This dampening is likely attributable to the adoption of more conservative trade strategies by countries when geopolitical risks intensify. For instance, nations may expand strategic grain reserves, curtail international trade, or introduce other measures to secure stable domestic grain supplies. Such responses tend to reduce transaction volumes in international grain markets, thereby weakening market interactions and limiting risk transmission. In addition, geopolitical risk exerts a negative influence on the net spillover of soybeans in both domestic and international markets, whereas the net spillover of wheat is positively affected. No significant effect is observed for the net spillover of corn. The quantile regression (QR) estimates further corroborate the ordinary least squares (OLS) findings and offer a more nuanced account of how geopolitical risk shapes grain net spillovers across different points of the distribution.

**TABLE 2:** Impact of China’s geopolitical risk on total and net spillovers of grain risk.

Variables	OLS	QR25	QR50	QR75
TCI	−3.371***	−3.837***	−3.834***	−3.673***
	(0.409)	(0.776)	(0.645)	(0.377)
Net CCORN	0.920	4.021***	2.804	−1.011
	(0.764)	(0.839)	(1.743)	(2.281)
Net CSOYBEANS	−0.912*	0.236	0.517	−1.361**
	(0.535)	(0.557)	(1.164)	(0.619)
Net CWHEAT	2.157***	2.347***	2.205***	2.014***
	(0.454)	(0.552)	(0.827)	(0.610)
Net WCORN	0.212	1.848*	−0.448	−2.078***
	(0.495)	(1.012)	(1.192)	(0.506)
Net WSOYBEANS	−4.837***	−3.368***	−6.837***	−5.322***
	(0.724)	(0.832)	(1.712)	(0.907)
Net WWHEAT	2.460***	3.501***	1.566***	0.990***
	(0.266)	(0.923)	(0.384)	(0.245)

***, **, * indicate significance at the 1%, 5%, and 10% levels, respectively. The values within the parentheses represent the standard errors.

The opposing net spillover effects of geopolitical risk on wheat and soybeans stem from their distinct policy weights and supply chain configurations. For wheat, as a core staple underpinning China’s food security, geopolitical tensions promptly evoke a “food security first” policy response. When risks escalate, for instance during the Russia Ukraine conflict, the Chinese government reinforces strategic reserves and raises minimum purchase prices, sending strong signals of policy intervention to the market. This uncertainty, stemming from policy interventions, not only shields wheat from external shocks but also amplifies its price spillovers to other crops, ultimately yielding a positive net spillover effect. In essence, geopolitical shocks render wheat a net transmitter of policy driven volatility. For soybeans, which are mainly used for animal feed and edible oil, the underlying economic logic is markedly different. Geopolitical disruptions, such as trade wars or shipping blockades, primarily raise freight rates, insurance costs, and exchange rate volatility for importers. These elevated transaction costs are directly absorbed by domestic crushing industries, making the domestic soybean market a net receiver of external cost push shocks. Moreover, given China’s flexibility to switch among suppliers (for example, the United States, Brazil, and Argentina), geopolitical risks tend to accelerate trade diversion rather than pass through to domestic prices. Consequently, the domestic soybean market is more vulnerable to imported risks than capable of exporting them, leading to a negative net spillover effect.

The effect of geopolitical risk on net spillovers is markedly heterogeneous across grain markets. An escalation of geopolitical risk exerts a negative influence on net spillovers in both international and domestic soybean markets, while its impact on international and domestic corn markets remains statistically non-significant. In contrast, geopolitical risk has a significantly positive effect on the net spillovers of both international and domestic wheat markets. This finding suggests that the wheat market, as a core supplier of staple food for human consumption, displays greater sensitivity to political events relative to other grain markets, thereby amplifying its net spillover levels.

#### 4.3.2 Equidistant impulse responses of grain risk spillovers

**FIGURE 6:**
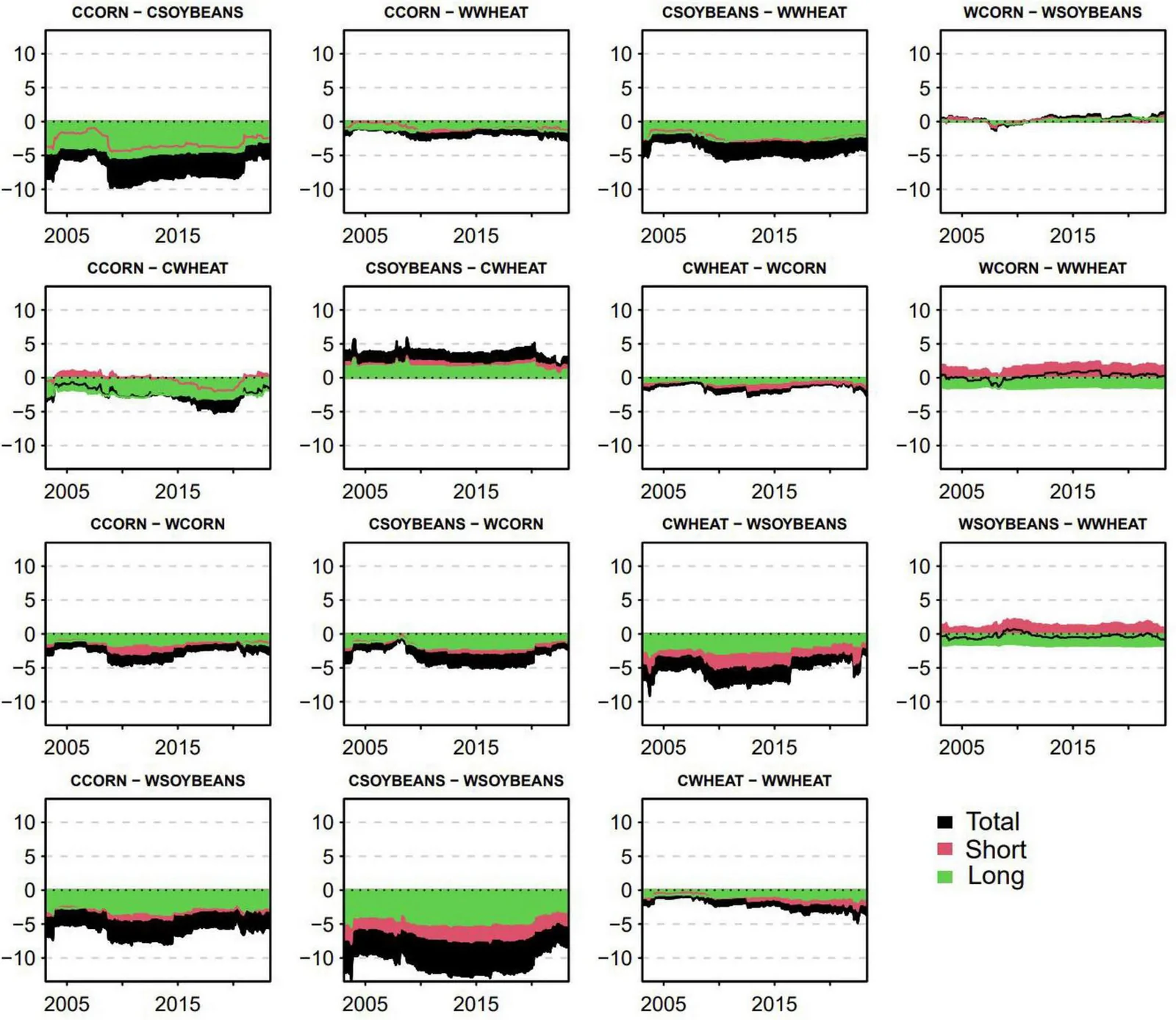
Net pairwise spillover.

This section employs the Time-Varying Parameter Vector Autoregression (TVP-VAR) model to investigate how geopolitical risks influence the spillover of grain market risks. Figure 6 reports the impulse responses of grain risk spillovers to global geopolitical risk (GPR), China’s geopolitical risk (GPRC), geopolitical threats (GPRT), and geopolitical actions (GPRA) at one-, two-, and four-period-ahead horizons. The evolving relationship between the geopolitical risk indices and grain risk spillovers across these forecast horizons reveals pronounced time variation in both the spillovers and their drivers. Moreover, the broadly consistent patterns observed across different forecast horizons confirm the robustness of the model estimates. Overall, the effects of alternative geopolitical risk indices on grain risk spillovers differ substantially.

The upper left subplot of Figure 6 illustrates the time-varying influence of global geopolitical risk (GPR) on grain risk spillovers, measured by the total connectedness index (TCI). Since 2003, this impact has fluctuated markedly, recording a trough with a positive impulse response in 2008, subsequently shifting into negative territory while oscillating at high frequency. The effect surged to a positive peak in the first half of 2016, then dropped sharply to a trough later that year. After 2019, the amplitude of these fluctuations narrowed, contributing to a pronounced reduction in grain risk spillovers. The impact of global geopolitical risks at different lag periods shows no significant difference on grain risk spillovers.

The bottom-left subplot depicts the time-varying impact of China’s geopolitical risk (GPRC) on grain risk spillovers, as measured by the total connectedness index (TCI). The trajectory of this impact broadly follows that of global geopolitical risk, yet notable differences emerge across forecast horizons. Specifically, at the two- and four-period-ahead horizons, the impulse responses remain positive and consistent during the periods 2007–2008 and after 2020, whereas negative responses prevail in the intervening years. The one-period-ahead response is markedly subdued and consistently negative. Compared with the influence of global geopolitical risk, the effect of China-specific geopolitical risk on grain spillovers is more attenuated. This attenuation stems largely from the fact that China’s geopolitical risks constitute a subset of global geopolitical risks, which encompass a broader set of events and consequently generate stronger impacts.

The top-right panel of Figure 6 illustrates the time-varying influence of geopolitical actions (GPRA) on grain risk spillovers, as captured by the total connectedness index (TCI). Between 2003 and 2012, GPRA exerted a markedly negative effect on grain risk spillovers, reaching a trough in the latter half of 2008, but the impulse response grew more volatile around 2012 and subsequently turned positive. After 2019, the impulse responses gradually attenuated and settled at a negative level.

The bottom-right panel depicts the time-varying impact of geopolitical threats (GPRT) on TCI. The response pattern associated with GPRT displays a notable counteracting tendency relative to that of GPRA. Pronounced positive effects are evident between 2007 and 2012 and again from 2015 to 2017, with a dip to a low point in the first half of 2012, while in the remaining periods the responses hover within a narrow band around the zero line.

The divergent effects of geopolitical threats and actions on grain risk spillovers are rooted in their structural and qualitative distinctions. Geopolitical threats capture latent sources of instability, such as political crises or military conflicts, which tend to foster unstable market expectations and amplify grain price volatility. By contrast, geopolitical actions, including trade agreements or economic sanctions, typically constitute premeditated policy measures that impose more direct and definitive impacts on grain markets, often contributing to price stabilization or declines. These contrasting mechanisms explain why the two forms of geopolitical risk influence grain market spillovers in markedly different directions.

#### 4.3.3 Time-specific impulse responses of grain risk spillovers

This study examines four critical episodes of heightened geopolitical risk: the Iraq Oil Crisis (April 2003), the Paris Terror Attacks (November 2015), the Escalation of the US-China Trade Conflict (May 2018), and the Russia-Ukraine War (March 2022). The impacts of geopolitical risk on grain market risk spillovers are simulated for these specific junctures. Overall, the magnitude, speed, and persistence of these impacts vary markedly across the four periods. During the 2003 Iraq Oil Crisis, global geopolitical risk (GPR), China’s geopolitical risk (GPRC), and geopolitical actions (GPRA) all generated strongly negative responses, which gradually receded toward the baseline after two periods, although the GPRC response remained closer to the baseline. In the 2015 Paris Terror Attacks, the effect of GPR was negligible and nearly zero, while both geopolitical threats (GPRT) and GPRA yielded positive impulse responses, with GPRT displaying greater volatility over the first four periods. During the 2018 Escalation of the US-China Trade Conflict, GPR and GPRA both exerted substantial influences on grain spillovers, but in opposite directions: GPR produced a positive response, whereas GPRA produced a negative one. GPRC and GPRT initially registered negative impacts, yet after three periods GPRT shifted to a promotive role. Amid the 2022 Russia-Ukraine War, all four geopolitical indices initially exerted strong negative effects on grain spillovers; however, the response of GPRC reversed from negative to positive following the first period. These divergent response patterns illustrate the multifaceted and context-dependent ways in which different forms of geopolitical risk shape grain market spillovers, determined largely by the specific nature and global repercussions of each geopolitical event.

## 5 Discussion

This study provides empirical evidence on the dynamic price linkages, risk spillovers, and the role of geopolitical risk in China’s grain markets. Our findings yield several implications that both corroborate and extend the existing literature.

The time-varying correlation between domestic and international grain prices, particularly the dominant short-run nature, reinforces the earlier observations of Engle (48), Diebold and Yilmaz (31) regarding volatility transmission in commodity markets. However, we find that the magnitude of correlation differs substantially across crop types, with corn exhibiting the highest sensitivity to international price movements. This result contrasts with the common assumption that staple grains (wheat) are most integrated globally (52). A plausible explanation lies in China’s import quota system for wheat and corn, which partially insulates wheat from external shocks, while corn imports, though quota-restricted, have grown more elastic due to rising feed demand. This nuance highlights the importance of disaggregating grain varieties when assessing market integration, a point often overlooked in aggregate studies.

Our static and dynamic spillover analysis confirms that the international grain market acts as the primary net transmitter of price risks, consistent with the findings of Hu et al. (29), Liu et al. (30) on oil and commodity markets. Notably, the net spillover of international soybeans (22.44%) far exceeds that of corn and wheat, which aligns with the high import dependence of China on soybeans. This result extends the work of published paper (53–55) by demonstrating that the direction and intensity of spillovers are not uniform across grains but are closely tied to each crop’s trade policy and demand structure. The pairwise spillover between domestic corn and soybeans also suggests that domestic markets are interconnected, implying that policy interventions targeting one crop may have unintended ripple effects on others.

TVP-VAR results reveal that geopolitical risk exerts heterogeneous and time-varying effects on grain spillovers. These findings are broadly in line with the event-study evidence of published paper (42, 56), but they go further by quantifying the dynamic response at different forecast horizons. Interestingly, the Russia-Ukraine conflict produced a sharp negative impulse on spillovers, which at first glance seems contradictory to the literature that typically associates conflicts with heightened volatility (57, 58). Over a longer horizon, however, the indirect cost channel via fertilizer prices may amplify spillovers, as suggested by our case analysis.

Overall, our findings underscore that geopolitical risk does not merely elevate price levels but also alters the transmission structure of risks across markets. This insight contributes to the growing literature on uncertainty and commodity markets by highlighting the differential roles of threats versus acts, and the conditional nature of their impacts (59, 60). For China, a major importer, the results imply that diversifying import sources and strengthening domestic reserve mechanisms are critical, a policy implication consistent with the recommendations of Neik et al. (61), Li et al. (62) for managing geopolitical exposure. While our analysis offers novel quantitative evidence, several limitations should be acknowledged. The DCC-GARCH and GFEVD models assume linearity in the conditional correlations, which may not fully capture non-linear dynamics during extreme events. Additionally, our geopolitical risk index is based on newspaper coverage, which may reflect media sentiment as much as actual risk. Future research could employ alternative measures, such as geopolitical distance or supply chain disruption indices, to further validate our findings.

## 6 The case of geopolitical risks impacting China’s grain imports

Russia, Ukraine, and the United States have long occupied pivotal positions in global agricultural trade and represent China’s principal sources of agricultural imports from Europe and the Americas, respectively. For instance, the Russia-Ukraine conflict led to a 43.3% decrease in China’s agricultural imports from Ukraine in 2022, while the Sino-U.S. trade friction resulted in a 42.14% reduction in imports from the U.S. in 2019 compared to 2017. In 2021, the combined wheat exports of these two countries accounted for more than 25 percent of the global total, while Ukraine alone supplied nearly 30 percent of China’s corn imports. The United States, for its part, has historically dominated China’s import markets for soybeans and cotton. From 2001 to 2017, it remained China’s largest supplier of agricultural products, consistently contributing over 20 percent of China’s total agricultural imports. Two major geopolitical events, however, have severely disrupted China’s agricultural import channels: the Russia–Ukraine conflict that erupted in 2022 and the escalating Sino–US trade friction that began in 2018 and intensified through 2025.

The outbreak of the Russia–Ukraine conflict in 2022 sent the geopolitical risk indices of both nations soaring and dealt a heavy blow to agricultural production and trade. The impact direction and magnitude of this event on the grain market are highly consistent with the impulse response results identified by the TVP-VAR model at the time point of March 2022 in the previous text. In Ukraine, damaged agricultural infrastructure and the blockade of trade ports sharply curtailed grain production and export capacity. Russia, subjected to Western sanctions, also faced restrictions on its agricultural exports. These disruptions jointly exacerbated the global imbalance between food supply and demand. For China, the conflict directly reduced the scale of agricultural imports from Ukraine: in 2022, China’s total agricultural imports from Ukraine fell to USD 2.97 billion, a year-on-year decline of 43.3 percent. Concurrently, Russia’s ban on fertilizer exports, imposed in response to sanctions, triggered a global shortage of agricultural inputs such as fertilizers and drove up their prices, further squeezing China’s imports of these essential inputs and raising domestic agricultural production costs. It is particularly worth noting that the correlation coefficient between domestic and foreign wheat prices revealed by the DCC-GARCH model exhibited a significant abnormal fluctuation between March and April 2022 (as shown in the middle subgraph of Figure 3), precisely coinciding with the critical time window when Russia and Ukraine (collectively accounting for over 25% of global wheat exports) suspended their exports due to the conflict. This fact corroborates the high sensitivity of the wheat market identified in the model to geopolitical events, and also concretizes the abstract correlation coefficient into a perceivable real impact. In the parallel case of Sino–US trade friction, agricultural products became the central arena of tariff retaliation. The United States imposed three rounds of tariff increases covering virtually all categories of agricultural goods, and China responded with countervailing tariffs on 517 agricultural items, including soybeans and grains. This tit-for-tat escalation caused China’s agricultural imports from the United States to drop by 42.14 percent in 2019 compared with 2017, reverting to a scale roughly equivalent to that observed in 2009. When tariff conflicts reignited in 2025, the cumulative US tariff rate on Chinese goods soared to 145 percent, and China implemented proportional countermeasures. The resulting high tariffs on both sides led to a steep collapse in US agricultural exports to China. Taken together, whether in the form of regional military conflict, as epitomized by the Russia–Ukraine war, or great-power rivalry, as illustrated by the Sino–US trade friction, geopolitical risks have generated powerful disturbances to China’s agricultural imports, underscoring the acute vulnerability of agricultural import security to geopolitical shocks.

Based on the typical cases mentioned above and the results obtained from Chapter 4, the following regular findings can be summarized: (1) The positioning of the international grain market as the main channel for risk transmission has been strongly confirmed at the factual level. Whether it is the disruption of the Black Sea trade route caused by the Russia-Ukraine conflict, or the tariff barriers resulting from the Sino-US trade friction, the primary impact on China’s food security is precisely reflected in the sudden narrowing of import sources. This precisely corroborates the conclusion drawn earlier based on the generalized prediction error variance decomposition (GFEVD), which stated that “the risk in the international market is exported outward by 128.52%.” (2) The heterogeneity of grain varieties is also significantly manifested at the typical factual level. Specifically, the impact of the Russia-Ukraine conflict on the wheat market was much stronger than that on the corn and soybean markets, while the influence of the Sino-US trade friction was mainly concentrated in the soybean import sector. This pattern forms a rigorous confirmation relationship with the abnormal fluctuation of the wheat correlation coefficient identified by the DCC-GARCH model, as well as the highest net spillover level (22.44%) of the soybean market in the GFEVD results. (3) The stylized facts presented above provide strong empirical support for the predominantly short-term nature of geopolitical shocks. In the cases examined in this study, the direct impact of geopolitical events on grain trade and prices was concentrated within a window of 1–6 months following the occurrence of the event. The longer-term effects were transmitted mainly through indirect channels, most notably by driving up the prices of agricultural inputs such as fertilizers and thereby raising agricultural production costs. This temporal pattern is dynamically consistent with the impulse response profiles obtained from the TVP-VAR model, which indicate that the impact attenuates gradually after 1–2 periods.

## 7 Conclusion, policy recommendations, and limitation

### 7.1 Conclusion

This study investigates food security by applying a set of analytical methods that include the DCC-GARCH model, generalized forecast error variance decomposition, and the TVP-VAR approach. Adopting a framework that traces the transmission from international food price shocks to grain market risk spillovers and onward to the impacts of geopolitical risk, we empirically examine how international price movements are dynamically transmitted to domestic markets, identify the evolving patterns of grain market risk spillovers, and assess the influence of geopolitical uncertainty on these spillovers. The main findings are summarized as follows.

(1) The dynamic correlations between international and domestic food prices differ across grain varieties and exhibit pronounced time variation, with effects predominantly concentrated in the short run. Domestic corn prices exhibit a pronounced V-shaped correlation trajectory with international prices, which we attribute primarily to the 2016 abolition of China’s temporary corn reserve policy and the transition to a market-based procurement system. This institutional reform exposed domestic corn prices to global market forces, with geopolitical shocks subsequently superimposing short-term volatility onto this longer-term structural trend. Soybean prices experienced considerable fluctuation during the 2008–2010 global food crisis but remained relatively stable in other periods. These results underscore that food price regulation should account not only for domestic market dynamics but also for the interconnections and contagion risks that link domestic and international markets.

(2) The total dynamic connectedness of the grain market is estimated at 245.11 percent, with within-market risk spillovers persistently outweighing cross-market spillovers. Both domestic and international wheat markets are most strongly influenced by their own lagged dynamics. The spillover structure exhibits clear asymmetry, as international grain markets generate the largest spillovers and act as net transmitters of price shocks. The most substantial net pairwise spillovers are observed between domestic corn and soybeans, and between domestic and international soybeans. Domestic soybean prices are affected by international soybean and wheat prices, and in turn transmit shocks to domestic wheat prices.

(3) The impact of geopolitical risk on grain market spillovers varies markedly across major geopolitical episodes, including the 2003 Iraq Oil Crisis, the 2015 Paris Terror Attacks, the 2018 escalation of the US-China trade conflict, and the 2022 Russia-Ukraine War. During the 2003 Iraq Oil Crisis, geopolitical risk produced a strong negative impact that gradually receded toward zero after two periods. The geopolitical risk associated with the Paris Terror Attacks exerted a comparatively weak influence on grain spillovers. In the case of the US-China trade conflict escalation, grain risk spillovers were positively affected by global geopolitical risk, whereas the Russia-Ukraine War generated substantial negative impacts. These contrasting patterns highlight the context-dependent nature of geopolitical risk transmission in grain markets.

(4) Empirical case studies further reveal that geopolitical risks pose a threat to China’s food security through two main channels: Firstly, they directly impact the stability of import sources. For instance, the conflict between Russia and Ukraine led to a 43.3% decrease in China’s total agricultural imports from Ukraine in 2022, and the trade friction between China and the United States resulted in a 42.14% reduction in China’s agricultural imports from the United States in 2019 compared to 2017. Secondly, they increase the prices of agricultural production materials such as fertilizers, thereby indirectly raising the production costs of domestic agriculture. The combination of these two transmission mechanisms highlights the vulnerability of China, as a major agricultural importer, in the complex geopolitical landscape.

### 7.2 Policy recommendations

Based on the preceding findings, the following policy recommendations are advanced. First, as efforts to develop a more open, competitive, and efficient food market continue, the linkage between international and domestic food prices will intensify. It is therefore imperative to strengthen real-time monitoring of international food price dynamics, establish integrated mechanisms for food price regulation and food security protection, and mitigate the transmission of severe international market turbulence to domestic food markets. Second, institutional capacities for managing geopolitical risk should be reinforced by constructing robust risk early-warning and emergency response systems. Diversifying import channels is equally critical to safeguarding the security of international food supplies and preserving market stability during episodes of geopolitical disruption. Third, international agricultural cooperation and exchanges should be deepened. Through bilateral and multilateral frameworks encompassing agricultural technology transfer, investment cooperation, and agri-food trade, countries can jointly address instabilities in food markets and mitigate the potential threats that geopolitical risks pose to food supply chains. Strengthened collaboration with international agricultural organizations is also essential for the co-development of rules and standards governing global food markets, thereby fostering fair, transparent, and stable market operations.

### 7.3 Limitation

Several limitations of this study should be acknowledged. First, our GFEVD analysis employs a binary domestic-versus-international framework that does not capture the multipolar structure of China’s soybean imports, particularly the roles of Brazil and Argentina as alternative suppliers. The direction and magnitude of soybean net spillovers may therefore be overestimated during trade frictions when import diversion occurs. Second, our geopolitical risk indices are based on newspaper coverage, which may reflect media sentiment as much as actual geopolitical events. Third, the DCC-GARCH and GFEVD models assume linearity in conditional correlations, which may not fully capture non-linear dynamics during extreme episodes. Future research could address these limitations by employing tripartite network analysis, alternative geopolitical risk measures, and non-linear modeling approaches. Finally, while we acknowledge the critical roles of China’s tariff-rate quota (TRQ) system and minimum purchase price (MPP) policy in shaping price transmission, a full institutional analysis of these policies’ time-varying effects is beyond the scope of this study. Future research could combine our quantitative framework with detailed policy coding to systematically assess how adjustments in quota volumes, minimum price levels, and reserve releases moderate geopolitical spillovers.

## Data Availability

Publicly available datasets were analyzed in this study. This study primarily uses the global and China-specific Geopolitical Risk Index, the Geopolitical Threats (GPRT) Index, and the Geopolitical Acts (GPRA) Index. Seasonal adjustments to the geopolitical risk indices were made using the Census X12 method. Data were sourced from the Economic Policy Uncertainty Database (http://www.policyuncertainty.com/gpr.html).

## Footnotes

1
http://www.policyuncertainty.com/gpr.html

## References

[1] Zhou P Yang S Wu X Shen Y. Calculation of regional agricultural production efficiency and empirical analysis of its influencing factors-based on DEA-CCR model and Tobit model. *J Comp Methods Sci Eng.* (2022) 22:109–22. 10.3233/JCM-215590

[2] Shen Y Guo X Zhang X. Digital financial inclusion, land transfer, and agricultural green total factor productivity. *Sustainability.* (2023) 15:6436. 10.3390/su15086436

[3] Li S Zhang Z Xu J Han J Zhuang H Cui X et al. Global food security under a sustainability perspective: assessment, challenges, and strategies. *Geography Sustainabil.* (2026) 7:100495. 10.1016/j.geosus.2026.100495

[4] Wei D. How does agricultural technological progress achieve synergistic growth of food security and farmers’ income: a case study from China. *Front Sustain Food Syst.* (2026) 10:1799657. 10.3389/fsufs.2026.1799657

[5] Gahamanyi TN Tchouassi G. Is food security impacted by price dynamics? proof from African nations. *J Agriculture Food Res.* (2025) 22:102063. 10.1016/j.jafr.2025.102063

[6] Che M Zhu Z Wen Y Liu Y. International food price swings and their consequences for the Chinese economy. *Econ Modell.* (2026) 155:107400. 10.1016/j.econmod.2025.107400

[7] Liu K Yamazaki M Koike A Mu Y. Corn trade simulations of China: reduction in tariffs versus expansion in tariff-rate quotas. *J Econ Stud.* (2022) 49:1284–303. 10.1108/JES-08-2021-0380

[8] Dorosh PA Minot N Rashid S. Food price stabilization: theory and lessons from experience. *Food Policy.* (2025) 137:102945. 10.1016/j.foodpol.2025.102945

[9] Li Q Yang J Yang X Mu E. Decompose food price disparities in China: evidence from wholesale markets. *Food Policy.* (2025) 131:102817. 10.1016/j.foodpol.2025.102817

[10] Hamilton JD. Oil and the macroeconomy since World War II. *J Polit Econ.* (1983) 91:228–48. 10.1086/261140

[11] Bremmer I. The end of the free market: who wins the war between states and corporations? *Eur View.* (2010) 9:249–52. 10.1007/s12290-010-0129-z

[12] Jensen N. Political risk, democratic institutions, and foreign direct investment. *J Polit.* (2008) 70:1040–52. 10.1017/s0022381608081048

[13] Davis CL. *Why Adjudicate? Enforcing Trade Rules in the WTO.* Princeton, NJ: Princeton University Press (2012). 10.23943/princeton/9780691152752.001.0001

[14] Kobrin SJ. Political risk: a review and reconsideration. *J Intern Bus Stud.* (1979) 10:67–80. 10.1057/palgrave.jibs.8490631

[15] MacDonald K. *The Politics of Global Supply Chains.* Hoboken, NJ: John Wiley & Sons (2014).

[16] Hill C. *International Business: Competing in the Global Market Place.* New York, NY: McGraw-Hill/Irwin (2008). 10.1108/sd.2008.05624iae.001

[17] Minor MS. The demise of expropriation as an instrument of LDC policy 1980–1992. *J Intern Bus Stud.* (1994) 25:177–88. 10.1057/palgrave.jibs.8490850

[18] Engle RF. Autoregressive conditional heteroscedasticity with estimates of the variance of United Kingdom inflation. *Econometrica: J Econometric Soc.* (1982) 50:987–1007. 10.2307/1912773

[19] Johansen S. Estimation and hypothesis testing of cointegration vectors in Gaussian vector autoregressive models. *Econometrica: J Econ Soc.* (1991) 59:1551–80. 10.2307/2938278

[20] Forbes KJ Rigobon R. No contagion, only interdependence: measuring stock market comovements. *J Finance* (2002) 57:2223–61. 10.1111/0022-1082.00494

[21] Brunnermeier MK. Deciphering the liquidity and credit crunch 2007–2008. *J Econ Perspect.* (2009) 23:77–100. 10.1257/jep.23.1.7720052301

[22] Kyle AS. Continuous auctions and insider trading. *Econometrica: J Econ Soc.* (1985) 53:1315–35. 10.2307/1913210

[23] Guidolin M La Ferrara E. The economic effects of violent conflict: evidence from asset market reactions. *J Peace Res.* (2010) 47:671–84. 10.1177/0022343310381853

[24] Ramiah V Wallace D Veron JF Reddy K Elliott R. The effects of recent terrorist attacks on risk and return in commodity markets. *Energy Econ.* (2019) 77:13–22. 10.1016/j.eneco.2018.10.025

[25] Balcilar M Gupta R Pierdzioch C. Does uncertainty move the gold price? New evidence from a nonparametric causality-in-quantiles test. *Resour Policy*. (2016) 49:74–80. 10.1016/j.resourpol.2016.04.004

[26] Joëts M Mignon V Razafindrabe T. Does the volatility of commodity prices reflect macroeconomic uncertainty? *Energy Econ.* (2017) 68:313–26. 10.1016/j.eneco.2017.09.017

[27] Chiang TC. Geopolitical risk, economic policy uncertainty and asset returns in Chinese financial markets. *China Finance Rev Intern.* (2021) 11:474–501. 10.1108/CFRI-08-2020-0115

[28] Bouoiyour J Selmi R Hammoudeh S Wohar M. What are the categories of geopolitical risks that could drive oil prices higher? Acts or threats? *Energy Econ.* (2019) 84:104523. 10.1016/j.eneco.2019.104523

[29] Hu M Zhang D Ji Q Wei L. Macro factors and the realized volatility of commodities: a dynamic network analysis. *Resources Policy.* (2020) 68:101813. 10.1016/j.resourpol.2020.101813 34173417 PMC7409805

[30] Liu Y Han L Xu Y. The impact of geopolitical uncertainty on energy volatility. *Intern Rev Financial Anal.* (2021) 75:101743. 10.1016/j.irfa.2021.101743

[31] Diebold FX Yılmaz K. On the network topology of variance decompositions: measuring the connectedness of financial firms. *J Econom*. (2014) 182:119–34. 10.1016/j.jeconom.2014.04.012

[32] Wang W Bai R. Analysis of the impact of agricultural product tariff quota management reform on China’s food security and countermeasures. *J Changzhou Univ.* (2022) 23:9–18. 10.3969/j.issn.2095-042X.2022.02.002

[33] Zhang T Sun H Pan M. Research on the transmission mechanism of international and domestic grain market prices - Based on the local equilibrium model. *Contemp Econ.* (2012) 296:6–7. 10.3969/j.issn.1007-9378.2012.08.003

[34] Su S Huo X. Evaluation of state trading distortions in China’s grain import sector: an empirical analysis from the perspective of tariff quota allocation methods. *J Huazhong Agricultural Univ.* (2019) 139:33–43. 10.13300/j.cnki.hnwkxb.2019.01.005

[35] Zhang R Gao Y. Research on the price linkage relationship between wheat in domestic and foreign markets. *Price: Theory Pract.* (2016) 385:112–5. 10.19851/j.cnki.cn11-1010/f.2016.07.029

[36] Li X Han Y Fu W. Dynamic integration analysis of wheat markets in China under the background of price support policy. *Res Agricultural Modern.* (2018) 39:405–13. 10.13872/j.1000-0275.2018.0007

[37] Li J Li J Xiao Q Wu H Liu D. Premium procurement channel access and farm-gate prices: evidence from rice farmers in China. *Food Policy.* (2026) 142:103132. 10.1016/j.foodpol.2026.103132

[38] Gong B Guo Q. Influence of maize acquisition and storage policy reform on rural land rent in Jilin province. *J Arid Land Res Environ*. (2021) 35:8–14. 10.13448/j.cnki.jalre.2021.121

[39] Gong L Jia K Xiong X. How major geopolitical events affect tail risk contagion in global crude oil markets —evidence from the Russia-Ukraine conflict. *Intern Rev Econ Finance.* (2025) 103:104523. 10.1016/j.iref.2025.104523

[40] Chen D Yu X Pardo-Piñashca E. Dynamic effects of EU economic sanctions on the EU-Russian energy market: evidence on crude oil and natural gas. *Energy Policy.* (2026) 210:114996. 10.1016/j.enpol.2025.114996

[41] D’Souza A Jolliffe D. Conflict, food price shocks, and food insecurity: the experience of Afghan households. *Food Policy.* (2013) 42:32–47. 10.1016/j.foodpol.2013.06.007

[42] Saâdaoui F Jabeur SB Goodell JW. Causality of geopolitical risk on food prices: considering the Russo–Ukrainian conflict. *Finance Res Lett.* (2022) 49:103103. 10.1016/j.frl.2022.103103

[43] Wang Q Gu H. Geopolitical conflicts, fluctuations in commodity prices, and short-term capital flows. *J Modern Finance.* (2023) 28:52–64. 10.16529/j.cnki.11-4613/f.2023.05.008

[44] Zhu J Lu J Su W. Empirical analysis of global crude oil transportation network amid the Russia-Ukraine conflict. *J Trans Geography.* (2025) 127:104305. 10.1016/j.jtrangeo.2025.104305

[45] Alnafissa M Ghanem AM. Modeling the effects of the Russia-Ukraine conflict on Egyptian food security. *Front. Sustain. Food Syst.* (2026) 10:1651967. 10.3389/fsufs.2026.1651967

[46] Shi J Wu L. Analysis of the impact of geopolitical risks on China’s grain trade from the perspective of food security. *Food Sci Technol Econ.* (2023) 48:12–7. 10.16465/j.gste.cn431252ts.20230403

[47] Sun Z Dai Y. Research on the impact of geopolitical risks on the prices of China’s grain futures market :taking the futures price of soybean No. 1 as an example. *Northern Finance J.* (2025) 536:39–45. 10.16459/j.cnki.15-1370/f.2025.02.006

[48] Engle R. Dynamic conditional correlation: a simple class of multivariate generalized autoregressive conditional heteroskedasticity models. *J Bus Econ Statist.* (2002) 20:339–50. 10.1198/073500102288618487

[49] Caldara D Iacoviello M. Measuring geopolitical risk. *Am Econ Rev.* (2022) 112:1194–225. 10.1257/aer.20191823

[50] Chen L. Research on the dynamic impact effect of international grain prices on China’s soybean prices. *Statist Manag.* (2023) 38:109–17. 10.16722/j.issn.1674-537x.2023.07.002

[51] Peng S Chen X. Source country risks and the stability of China’s grain imports. *Food Econ Manag.* (2025) 3:24–36. 10.20067/j.cnki.swjjygl.2025.02.003

[52] Chen X Tongurai J. Price spillovers and interdependences in China’s agricultural commodity futures market: evidence from the US-China trade dispute. *Intern Rev Econ Finance.* (2024) 96:103579. 10.1016/j.iref.2024.103579

[53] Hu G Liu S Wu G Hu P Li R Chen L. Economic policy uncertainty, geopolitical risks, and the heterogeneity of commodity price fluctuations in China ——an empirical study based on TVP-SV-VAR model. *Resources Policy.* (2023) 85:104009. 10.1016/j.resourpol.2023.104009

[54] Qi C Meng C Yan S. Transmission effect of international grain prices on China’s grain prices. *Front Sustain Food Syst.* (2025) 9:1483424. 10.3389/fsufs.2025.1483424

[55] Larvoe N Mustapa MAC Ramzan ME Gil JM Kallas Z. Market connectedness and price spillovers in Ghanaian maize and yam markets: implications for food security. *Food Policy.* (2026) 142:103143. 10.1016/j.foodpol.2026.103143

[56] Karkowska R Urjasz S. Importance of geopolitical risk in volatility structure: new evidence from biofuels, crude oil, and grains commodity markets. *J Commodity Markets.* (2024) 36:100440. 10.1016/j.jcomm.2024.100440

[57] Abbas S Alnafrah I. Food security in Pakistan: investigating the spillover effect of Russia-Ukraine conflict. *Sustainable Futures.* (2024) 8:100300. 10.1016/j.sftr.2024.100300

[58] Urak F Bilgiç A Florkowski WJ Bozma G. Confluence of COVID-19 and the Russia-Ukraine conflict: effects on agricultural commodity prices and food security. *Borsa Istanbul Rev.* (2024) 24:506–19. 10.1016/j.bir.2024.02.008

[59] Laubsch J Smales LA Vo D. The influence of uncertainty on commodity futures returns and trading behaviour. *Quar Rev Econ Finance.* (2024) 98:101915. 10.1016/j.qref.2024.101915

[60] Chakradhar J Ganguly A Choudhury RN Jadhav P. Trade policy uncertainty and agricultural exports: the mitigating roles of RTA and liberal democracies. *Transnatl Corp Rev.* (2025) 17:200160. 10.1016/j.tncr.2025.200160

[61] Neik TX Siddique KHM Mayes S Edwards D Batley J Mabhaudhi T et al. Diversifying agrifood systems to ensure global food security following the Russia–Ukraine crisis. *Front Sustain Food Syst.* (2023) 7:1124640. 10.3389/fsufs.2023.1124640

[62] Li X Liu H Wang Z Chen H. Import diversification, market risk co-movement and Agri-food supply chain resilience. *China Econ Rev.* (2025) 93:102483. 10.1016/j.chieco.2025.102483

